# Development and application of SYBR Green I real-time PCR assay for the separate detection of subgroup J Avian leukosis virus and multiplex detection of avian leukosis virus subgroups A and B

**DOI:** 10.1186/s12985-015-0291-7

**Published:** 2015-04-03

**Authors:** Manman Dai, Min Feng, Di Liu, Weisheng Cao, Ming Liao

**Affiliations:** College of Veterinary Medicine, South China Agricultural University, Guangzhou, People’s Republic of China; Key Laboratory of Veterinary Vaccine Innovation of the Ministry of Agriculture, Guangzhou, People’s Republic of China; College of Animal Science, South China Agricultural University, Guangzhou, People’s Republic of China

**Keywords:** Avian leukosis virus, Subgroups, Fluorescent quantitative and real-time PCR, SYBR Green I

## Abstract

**Background:**

Subgroup A, B, and J ALVs are the most prevalent avian leukosis virus (ALV). Our study attempted to develop two SYBR Green I-based real-time PCR (RT-PCR) assays for specific detection of ALV subgroup J (ALV-J) and multiplex detection of ALV subgroups A and B (ALV-A/B), respectively.

**Results:**

The two assays showed high specificity for ALV-J and ALV-A/B and the sensitivity of the two assays was at least 100 times higher than that of the routine PCR assay. The minimum virus detection limit of virus culture, routine PCR and real-time PCR for detection of ALV-A strain was 10^3^ TCID_50_ units, 10^2^ TCID_50_ units and fewer than 10 TCID_50_ units, respectively. In addition, the coefficients of variation for intra- and inter-assay were both less than 5%. Forty clinical plasma samples were evaluated by real-time PCR, routine PCR, and virus culture with positive rates of 80% (32/40), 72.5% (29/40) and 62.5% (25/40), respectively. When the assay for detection of ALV-J was used to quantify the viral load of various organ tissues in chicken inoculated by ALV-J strains CHN06 and NX0101, the results exhibited that ALV-J genes could be detected in all organ tissues examined and the highest copies of ALV-J were mainly in heart and kidney samples at 30 weeks post-infection. Except in lung, the virus copies of CHN06 group were higher than that of NX0101 group in various organ tissues.

**Conclusions:**

The SYBR Green I-based real-time RT-PCR assay provides a powerful tool for the detection of ALV and study of virus replication and infection.

## Background

Avian leukosis viruses (ALVs) belong to the genus *Alpharetrovirus* of the family *Retroviridae* and can induce different pathotypes of neoplastic diseases in birds including lymphoid and myeloid leukosis, resulting in severe economic losses in the poultry industry worldwide. Based on the variation in the nucleotide sequence of *gp85*, ALV strains from chickens have been classified into subgroups A, B, C, D, E, J, and the recently identified subgroup K [1-3]. It is noteworthy that subgroup A, B, and J are the most common pathogenic exogenous viruses. Subgroup J was first isolated in commercial broiler breeders with myelocytomatosis and has caused significant economic losses in the broiler industry because of increased tumor-induced mortality, decreased weight gain, serious immunosuppression and cost for eradication [4-6]. ALV A and B (ALV-A/B) mainly infect layer and breeder chickens leading to higher incidence of lymphoid leukosis (LL) or erythroblastosis (EB) in susceptible chickens and cause severe economic losses due to decreased productivity, including reduction in weight gain, egg production and breeding potential [7-9]. Currently, there are no effective vaccines or drugs against ALV. Therefore, early identification and removal of virus-shedding birds are key measurements in the control of exogenous ALV infections.

Different methods have been established for detecting exogenous ALV, including traditional virus isolation plus an antigen-capture enzyme-linked immunosorbent assay (ELISA) for group-specific p27 antigen of ALV, immunofluorescence assay (IFA), loop-mediated isothermal amplification (LAMP), and quantitative competitive reverse transcription PCR (QC-RT-PCR)[10-13]. However, each of these methods has limitations. For instance, ELISA and IFA are both time-consuming and quantitative data can’t be acquired by the current LAMP method. Although QC-RT-PCR can be quantitative, it is not a convenient method to use. Currently, a quantitative real-time PCR method based on a TaqMan probe is used for rapid, sensitive and accurate diagnosis of ALV-J and ALV-A/B [14,15]. Though real-time PCR using fluorescent probe detection is specific, it is relatively expensive and difficult to design specific probe. In contrast, the SYBR Green I real-time PCR assay is relatively inexpensive and more convenient in that it does not require a specific probe but rather requires specific primers.

The current study was undertaken to develop a simple, inexpensive, sensitive and specific SYBR Green I quantitative real-time PCR method for separate detection of ALV-J and multiplex detection of ALV-A/B, respectively. The real-time PCR method was further applied to evaluate clinical plasma samples and investigate the distribution of ALV-J in poultry organs at the later stage of infection.

## Results

### Standard curve for SYBR GreenIreal-time PCR

**Figure 1:**
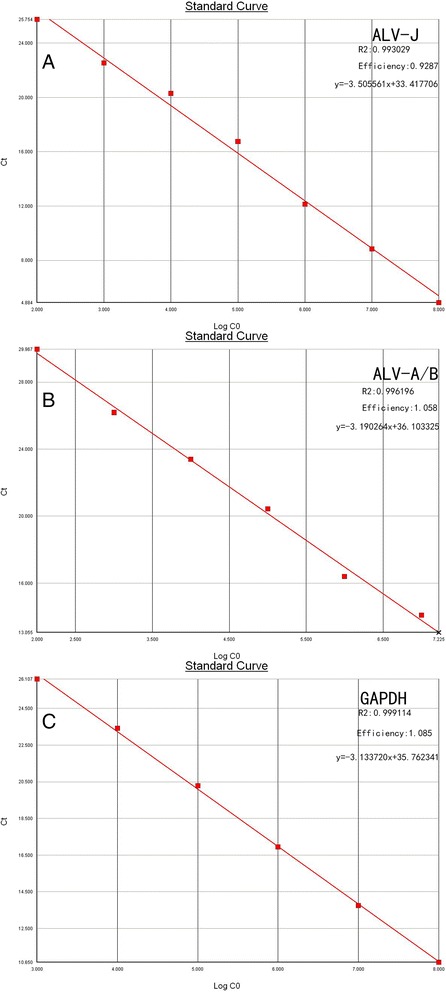
**Standard curve of real-time PCR for the detection of ALV-J, ALV-A/B, and GAPDH. (A)** ALV-J plasmid ranged from 10^2^ to10^8^ copies/μL, **(B)** ALV-A/B plasmid ranged from 10^2^ to10^8^ copies/μL, **(C)** GAPDH plasmid ranged from 10^3^ to10^8^ copies/μL.

Serial dilutions from 10^8^ - 10^2^ copies/μL of plasmid pMDA/B, and pMDJ and 10^8^ - 10^3^ copies/μL of plasmid pMDG were used to produce standard curves for the qRT-PCR. Threshold cycle (C_*T*_) values were plotted against the known copy numbers of the standard controls. The results showed that there was good correlation between copy number and C_*T*_ value of pMDA/B (R^2^ = 0.996196, efficiency = 1.058), pMDJ (R^2^ = 0.993029, efficiency =0.9287) and pMDG (R2 = 0.999114, efficiency = 1.085), respectively (Figure 1).

### Specificity of the SYBR GreenIreal-time PCR

**Figure 2:**
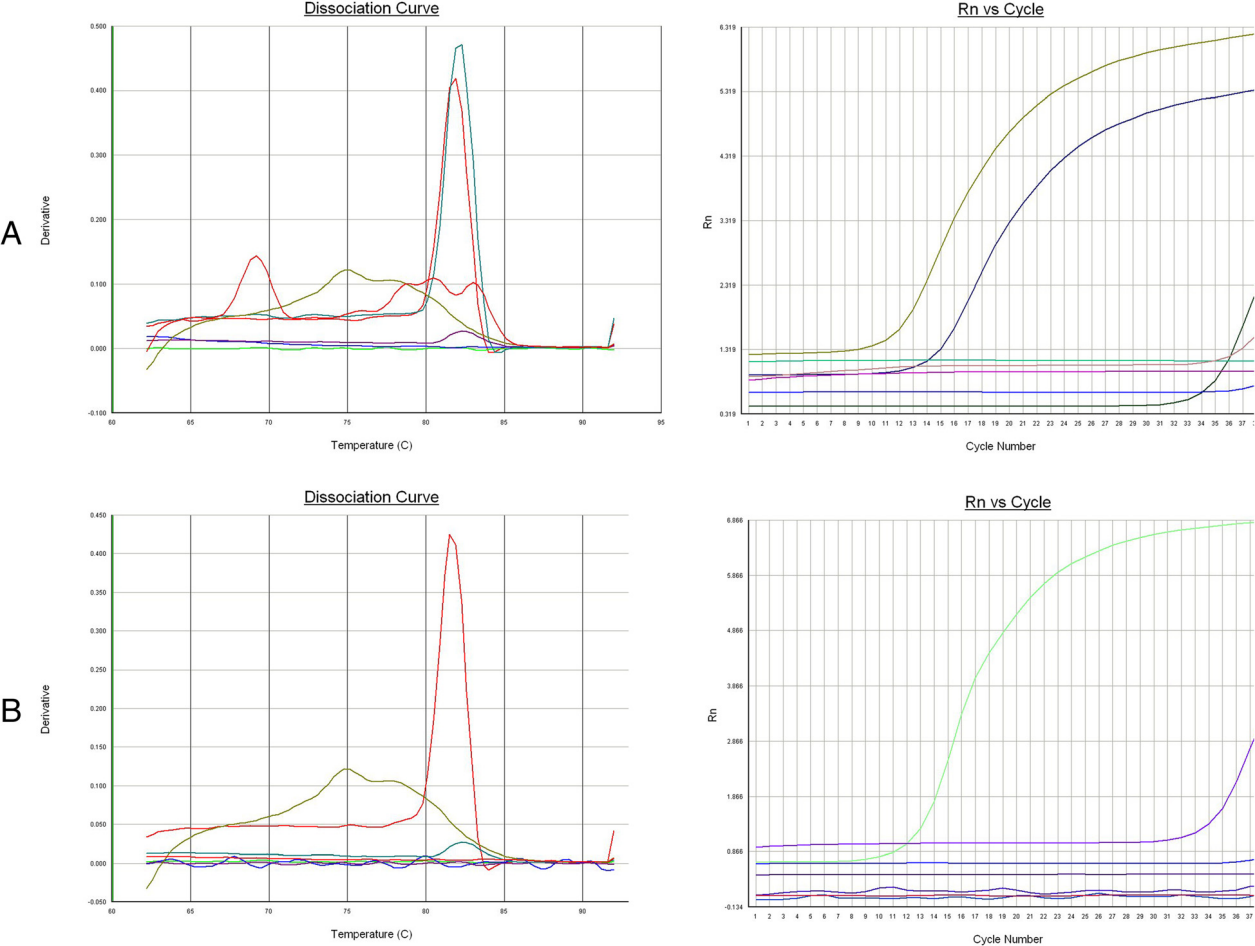
**Specificity of real-time PCR assay. (A)** The specific fluorescent signals were detected from cDNA of GD13-1 and CD08 strains. No cross-reactions were detected from AIV, NDV, ALV-J, ALV-E, DF-1. **(B)** The dissociation curves showed that the CHN06 strain were considered as positive and other samples were considered as negative.

Specific fluorescent signals were detected in the cDNA of ALV A subgroup strain GD13-1 and B subgroup strain CD08 (Figure 2). However, no fluorescent signals were detected in the cDNA of ALV J subgroup strain CHN06, AIV strain H9N2 , and NDV strain GM as well as genomic DNA of ALV E subgroup strain HN1301 by the duplex qRT-PCR. Similarly, the cDNA of ALV J subgroup strain CHN06 could be detected; however, there was no detection of fluorescent signals from the other virus samples, including ALV-A GD13-1, ALV-E HN1301, AIV H9N2 and NDV GM, suggesting a specificity of the real-time PCR for ALV-J.

### SYBR Green I Real-time PCR sensitivity compared to routine PCR

**Figure 3:**
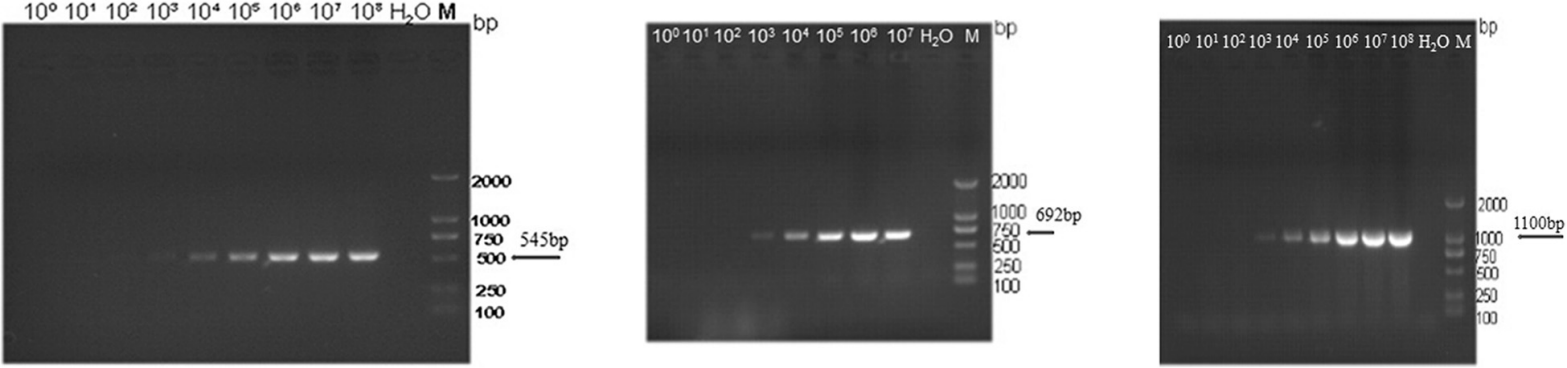
**The sensitivity of conventional PCR assay.** The 545 bp, 692 bp and 1.1 kb amplicons specific to ALV-J, ALV-A and ALV-B, respectively, were detected by routine PCR with the specific primers (Table 7). The detection limit was 1000 copies. Lane M, DNA marker DL-2000 (Dongsheng Biotech).

Detection limits for routine PCR with the H5/H7 primer pair, the H5/CAPA primer pair, and the BD-F/BD-R primer pair were 1.34 × 10^3^ copies, 1.6 × 10^3^ copies and 2.6 × 10^3^ copies, respectively (Figure 3). The detection limits of the real-time PCR for ALV-J, ALV-A and ALV-B were as low as 5.5 × 10^1^ copies, 1.1 × 10^1^ copies and 3.5 × 10^1^ copies respectively (data not shown). Therefore, the sensitivity of our real-time PCR assay was at least 100 times higher than that of the routine PCR assay.

### Reproducibility of SYBR Green I Real-time PCR

To assess the reproducibility of the RT-PCR assay, the C_*T*_ values for plasmids pMDA, pMDB, pMDJ, and pMDG (from 10^5^ to 10^7^ plasmid copies/μL) were detected in both inter-assays and intra-assays that were performed in triplicate. As demonstrated in Tables 1, 2, 3 and 4, coefficients of variation (CV) of C_*T*_ values were less than 5%. More specifically, the CV of intra- and inter-assays ranged from 0.104% ~ 1.000% and from 0.586% ~ 1.827% for the plasmid pMDJ, 0.569% ~ 1.007% and 0.285% ~ 2.52% for pMDA, 0.125% ~ 1.042 and 1.380% ~ 2.385% for pMDB, and 0.292% ~ 1.296% and 1.528% ~ 1.932% for pMDG, respectively.

**Table 1:** **Reproducibility of pMDJ**

**Reproducibility**	**No. of DNA copies**	**Ct (mean ± SD)**	**CV (%)**
intra- assay	5.5 × 10^5^	18.56 ± 0.147	0.792
	5.5 × 10^6^	15.32 ± 0.016	0.104
	5.5 × 10^7^	13.90 ± 0.139	1.000
interassay	5.5 × 10^5^	18.81 ± 0.233	1.239
	5.5 × 10^6^	15.05 ± 0.275	1.827
	5.5 × 10^7^	13.82 ± 0.081	0.586

**Table 2:** **Reproducibility of pMDA** ^**α**^

**Reproducibility**	**No. of DNA copies**	**Ct (mean ± SD)**	**CV (%)**
intra- assay	1.1 × 10^5^	19.94 ± 0.191	0.958
	1.1 × 10^6^	16.38 ± 0.165	1.007
	1.1 × 10^7^	13.36 ± 0.076	0.569
interassay	1.1 × 10^5^	20.00 ± 0.057	0.285
	1.1 × 10^6^	16.43 ± 0.133	0.809
	1.1 × 10^7^	13.76 ± 0.347	2.52

α: pMDA was obtained from GD13-1 cDNA with the primer of A/B-F and A/B-R.

**Table 3:** **Reproducibility of pMDB** ^**β**^

**Reproducibility**	**No. of DNA copies**	**Ct (mean ± SD)**	**CV (%)**
intra- assay	3.5 × 10^5^	17.18 ± 0.179	1.042
	3.5 × 10^6^	14.62 ± 0.066	0.451
	3.5 × 10^7^	13.59 ± 0.017	0.125
interassay	3.5 × 10^5^	16.73 ± 0.399	2.385
	3.5 × 10^6^	14.56 ± 0.326	2.239
	3.5 × 10^7^	13.41 ± 0.185	1.380

β: pMDB was obtained from CD08 cDNA with the primer of A/B-F and A/B-R.

**Table 4:** **Reproducibility of pMDG**

**Reproducibility**	**No. of DNA copies**	**Ct (mean ± SD)**	**CV (%)**
intra- assay	2.2 × 10^5^	20.77 ± 0.136	0.655
	2.2 × 10^6^	16.36 ± 0.212	1.296
	2.2 × 10^7^	14.37 ± 0.042	0.292
interassay	2.2 × 10^5^	20.34 ± 0.393	1.932
	2.2 × 10^6^	16.49 ± 0.264	1.601
	2.2 × 10^7^	14.40 ± 0.220	1.528

### Detection of plasma samples by three kinds of methods

A total of 40 blood samples suspected of ALV infection were simultaneously evaluated using the real-time PCR method , routine PCR, and virus culture isolation with p27 detection with positivity rates of 80% (32/40), 72.5% (29/40) and 62.5% (25/40), respectively (Table 5). Positive samples by both routine PCR and virus culture isolation with p27 detection were also positive by real-time PCR. Among those positive samples, there was one that was infected with both ALV-J and ALV-A.

**Table 5:** **Detection of ALV from clinical plasma samples**

**Method**	**Real-time PCR**		**Routine PCR**			**Virus isolation (** ***p27*** **detection)**
	**J-F/J-R**	**AB-F/AB-R**	**H5/H7**	**H5/CAPA**	**BD-F/BD-R**	
Positivity rate	77.5% (31/40)	5% (2/40)	70% (28/40)	5% (2/40)	0% (0/40)	62.5% (25/40)
Total Positivity rate	80% (32/40)		72.5% (29/40)			62.5% (25/40)

### The minimum virus detection limit

**Figure 4:**
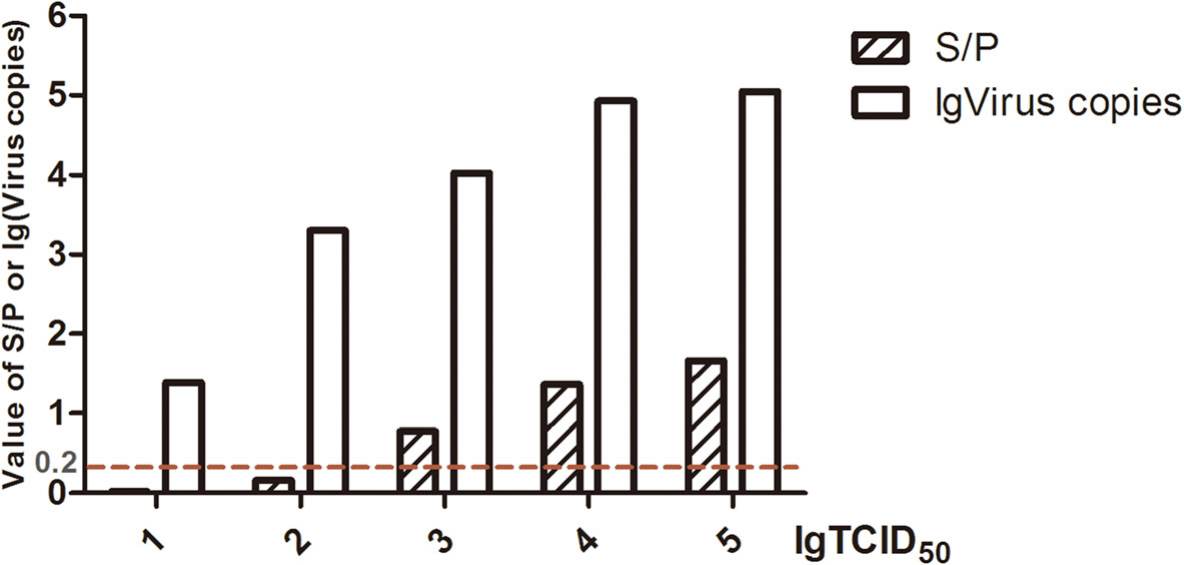
**The minimum virus detection limits of ELISA, routine PCR and real-time PCR.** The 692 bp amplicons to GD13-1 strain was detected by routine PCR (data not shown), and the S/P values and virus copies were detected by ELISA and real-time PCR, respectively.

The detection limits of the three methods were determined using dilutions (10^1^ to 10^5^ TCID_50_) of GD13-1 strain prepared in DMEM inoculated into DF-1 cells. The minimum virus detection limits of virus culture isolation, routine PCR, and real-time PCR were 10^3^ TCID_50_ units, 10^2^ TCID_50_ units (data not shown), and as low as 10 TCID_50_ units, respectively (Figure 4). Therefore, the minimum virus detection limit of real-time PCR was at least 10 times lower than that of the routine PCR, which was 10 times better than that of virus culture isolation with p27 detection. Both S/P values and virus copies increased constantly as the virus titer gradually rose.

### Viral load quantification assay in various organ tissues infected with ALV-J

**Figure 5:**
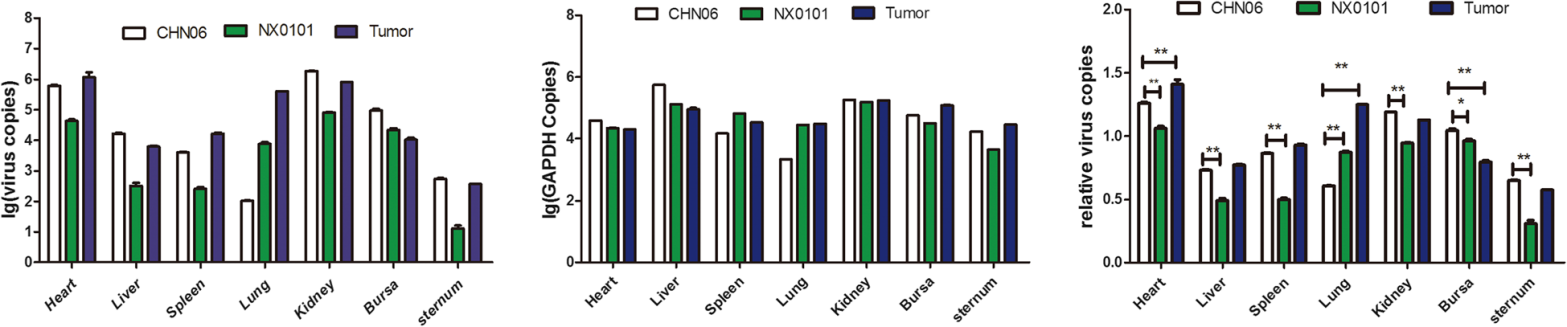
**The ALV-J gene copies, GAPDH copies and relative virus copies among different organs in CHN06 group, NX0101 group and Tumor case.** Statistical analysis was made using Two-way ANOVA in GraphPad Prism 5. *P < 0.01, **P < 0.001. Error bars indicate SEM.

Tissue samples of infected groups evaluated both by routine PCR and virus culture isolation with p27 antigen detection were all positive while control groups were all negative (data not shown). In addition, there were three cases with tumors, including two cases infected with NX0101 and one with CHN06. According to the standard curve formula: y = −3.505561x + 33.417706, the lg value of the virus copies in various organ tissues infected with ALV-J could be calculated by measuring the Ct value. At the same time, the lg value of the GAPDH copies in various organ tissues could be calculated by measuring the Ct value in accordance with the standard curve formula: y = −3.133720x + 35.762341.Data are obtained from the average of multifarious organ tissues of each group. To compare ALV-J gene copies among different organs in three groups, relative virus copies could be calculated by measuring quotient of the lg value of the virus copies divided by the lg value of the GAPDH copies. As Figure 5 showed, ALV-J genes could be detected in all organs of each group. In the CHN06 group, the heart, kidney and bursa had the highest viral gene load, liver and spleen had a medium viral load, and the lung and sternum had the lowest load. In NX0101 group, the copies of ALV-J were respectively highest in heart, higher in lung, kidney and bursa, and lowest in liver, spleen and sternum. In the tumor case, the heart, lung and kidney had the highest viral gene load; liver, spleen and bursa had a medium viral load, and the sternum had the lowest load. Except in the lung, the virus copies of CHN06 group were higher than that of NX0101 group in various organ tissues. From the above results, the highest copies of ALV-J were found in the heart and kidney.

## Discussion

Although the epidemic of ALV-A/B infection was under control in the modern-intensive chicken farms throughout the world by the 1980s, both ALV- A and ALV-B viruses are still isolated from chickens and wild birds in recent years in China [16-18]. More seriously, ALV-J isolates have frequently been recovered from broiler breeders and layer chickens in most parts of China during the past twenty years [19]. Lack of effective vaccines or drugs to prevent or treat ALV infections and the widespread distribution of ALV via vertical and/or horizontal transmission in chicken flocks, especial the local chicken breeds, have presented major challenges for the control and eradication of exogenous ALV; thus, it is necessary to be able to promptly identify and remove virus-shedding birds early to minimize the spread of congenital and contact infection.

ALV is a retrovirus, in which the RNA genome in the viral particles is reversely transcribed into a DNA form, i.e., the provirus, in the infected cells. Hence, detection of the virus by PCR amplification of the specific fragment within the genome is usually carried out. Classification of ALV subgroups is based on the gene sequence of the ALV *gp*85 gene. We designed primers for ALV-A/B and ALV-J, after aligning published *gp*85 sequences available by DNAStar. Furthermore, we found ALV-A/B and ALV-J primers shared no complementary reaction with other subgroups of ALV via primer-BLAST search. The specificity of the ALV-A/B duplex qRT-PCR was evaluated, and no specific dissociation curve was detected from ALV J and E subgroups as well as other common avian viruses, including AIV and NDV. Likewise, the specificity of the ALV-J real-time detection methods was examined, and the melting curve showed a single peak that could only be detected for ALV-J. The detection limits of the method were as low as 55 copies for ALV-J, 11 copies for ALV-A and 35 copies for ALV-B, which is at least 100 times more sensitive than that of the conventional PCR with a detection limit of 10^3^ copies. The variation coefficient of repeat tests for plasmids pMDA, pMDB, pMDJ and pMDG were all less than 5%.

The qRT-PCR assay was more effective than conventional PCR and virus culture isolation in the examination of 40 clinical samples, where three samples and seven samples were negative by conventional PCR and virus isolation tests, respectively, but they were positive by the qRT-PCR. DF-1 cells inoculated with viral loads ranging from 10^1^ to 10^5^ copies of GD13-1 strain were detected using three methods consisting of p27 detection, routine PCR and real-time PCR. The results demonstrated that the minimum virus detection limit of real-time PCR (as low as 10 TCID_50_ units) was at least 10 times lower than that of the routine PCR (10^2^ TCID_50_ units), which was 10 fold lower than that of virus isolation with p27 detection (10^3^ TCID_50_ units).

When real-time PCR is widely used to quantify viral genes, a host gene expressed steadily in a host cells or tissue samples as an internal control becomes one pivot point for calculating the copy number of specific viral genes. Zhou et al., used the *gp*85 gene and the chicken *β*-actin gene as PCR targets for viral gene and the host cell, respectively [15]. This allows the errors in RNA or DNA extraction in different samples to be minimized as a result of the use of an internal control host gene. In addition, the mRNA expression of target genes in different samples or different experimental groups can be compared. In this study, the host gene, GAPDH, was used as an internal control for calculating the copy number of ALV-J in various organ tissues. An animal experiment using SPF chickens infected with the CHN06 and the NX0101 strains was carried out to determine the extent of virus distribution and load of ALV-J in different organs in each group at 30 weeks post-infection by p27 antigen detection, routine PCR and real-time PCR methods. The results showed that various organ tissues of infected group were all positive by the three methods, indicating that the ALV-J was found in all detected organs at 30 weeks post-infection. As shown in Figure 5, virus copies of CHN06 group were higher than that of NX0101 group in various organ tissues except in lung. This result suggested that the replication rates in organ tissues of the respective ALV-J strains CHN06 and NX0101 associated with hemangioma and myelocytoma were different. Combined with previous experimental results that the replication rates in DF-1 cells of CHN06 strain were higher than NX0101 strain (data not shown), it was speculated that the number of virus copies is correlated to viral replication capacity. The difference of virus copies in lung between the CHN06 and NX0101 groups implied that tissue tropisms of the two strains exist. It was reported that env and LTR regions of ALV were responsible for distinctive tissue tropisms [20,21]. Therefore, we propose that the difference of virus copies in lung between CHN06 and NX0101 groups may be related to the diversity in the gene expression of env and LTR regions. In our study, the highest number of copies for ALV-J was in heart and kidney tissues at 30 weeks post-infection, during which the tumors were mainly formed. However, we didn’t study organs distributions of ALV-J at the other infective stages. It is also possible that the results of organs distributions may be associated with sampling time, strain, infective stage and individual variation.

Real-time RT-PCR methods with SYBR green and fluorescence probe have been widely used in the detection of many pathogenic microorganisms. Nevertheless, they share the common limitations of false-positive due to high sensitivity requiring no more than 10 viral copies in the samples for detection. Although TaqMan probes provide additional specificity relative to SYBR green, it is relatively more expensive and inconvenient for clinical and experimental detection. In contrast, SYBR green real-time RT-PCR method is low-cost and convenient for detection. Our study performed using both methods allowed to limit non-specific amplifications, assisted with specific primer designed for SYBR green real-time RT-PCR.

In conclusion, SYBR GreenIreal-time PCR assays for the separate detection of subgroup J avian leukosis virus and multiplex detection of avian leukosis virus subgroups A and B are specific, sensitive, inexpensive, rapid, and convenient. Moreover, this assay can be used to analyze viral load quantification in various organ tissues and explore the tissue tropisms of ALV strains. The developed real-time PCR methods will promote the detection of ALV and study of virus replication and infection.

## Materials and methods

### Ethics statement

None of the experiments in our study involved human participants. Forty plasma samples including twenty-five p27 antigen-positive samples and fifteen p27 antigen-negative samples were collected from forty yellow chickens of Guangdong province in China during an ALV epidemiological investigation conducted by our laboratory. The animal research obtained specific approval and guidance from South China Agriculture University’s Institutional Animal Care and Use Committee. Animal research in our study had also been approved by Guangdong Province Animal Disease Control Center.

### Virus and clinical plasma samples

ALV A subgroup strain GD13-1, ALV B subgroup strain CD08, and ALV J subgroup strain CHN06 were isolated and identified by our laboratory and each was proliferated in DF-1 cells for extraction of total cellular RNA followed by cDNA synthesis. The ALV J isolate NX0101 was a gift from Professor Zhizhong Cui of Shandong Agricultural University. The cDNA from Newcastle Disease virus (NDV) strain GM, Avian Influenza Virus (AIV) strain H9N2 and genomic DNA from ALV E subgroup strain HN1301 were maintained in our laboratory and used to check the specificity of the qRT-PCR assay. Forty plasma samples (including twenty-five p27 antigen-positive samples and fifteen p27 antigen-negative samples) collected from forty yellow chickens of Guangdong province in China during the ALV epidemiological investigation conducted by our laboratory were proliferated in DF-1 cells, which are known to be susceptible to exogenous ALV for 7 days [22]. After repeated freezing and thawing for three times, the DF-1 cells were centrifuged in 4°C at 2,000 g for 2 min. The supernatants were harvested and used to determine ALV p27 with a commercial ELISA kit (IDEXX, Inc., Westbrook, MA) and total cellular RNA was extracted from DF-1 cells with the RNAfast200 kit (Fastagen), followed by cDNA synthesis with the RevertAid First strand cDNA synthesis kit (Fermentas) according to the manufacturer’s instructions. The generated cDNA was then used for routine PCR and real-time PCR amplification.

### Cell infection

The DF1 cells grown to monolayer were digested with 0.25% trypsin (Gibco), and the cells were then adjusted to density of 2.0 × 10^5^ cells/mL in Dulbecco’s modified Eagle’s medium (DMEM) (Gibco) with 10% FBS (Gibco) in 6-well cell culture plates at 37°C and 5% CO_2_ until they reached approximately 80% confluence. Five different virus titers from 10^1^ TCID_50_ to 10^5^ TCID_50_ per 0.2 ml of ALV-A (GD13-1 strain) were inoculated per well in two 6-well cell culture plates containing DF-1 cells. Two wells on each plate served as the negative control. Each dilution of virus was performed in duplicate. After the inoculum was removed, maintenance medium containing DMEM with 2% FBS was added and the plates were incubated at 37°C and 5% CO2 for another 5 days. The supernatant fluid was then harvested for ALV p27 antigen detection, while total RNA and cDNA from the infected cells were extracted and synthesized as described above.

### Samples from ALV-J (CHN06 and NX0101 strains) infected SPF chickens

A total of 30 one-day-old specific-pathogen-free (SPF) egg laying types of chickens (Beijing Merial Vital laboratory Animal Technology Co., Ltd., Beijing, China) were randomly assigned to three study groups: CHN06 infected group, NX0101 infected group and control group with ten chickens per group. Infected groups were intraperitoneally inoculated at a dose of 0.2 mL (10^4.5^ TCID_50_ /0.2 mL). Control group was injected with DMEM media alone. All groups were reared separately in negative-pressure isolators. Heart, liver, spleen, lung, kidney, bursa, and sternum samples from each bird were collected at 30 weeks post-infection and stored at −80°C until further used. Tissue samples (0.2 g each) were homogenized in phosphate-buffered saline (PBS) and subsequently centrifuged at 1,4000 g for 5 min at 4°C after three rounds of continuous freeze-thawing. Supernatant was gathered and stored at −80°C. A portion of the supernatant was used for p27 antigen detection using an avian leukosis virus antigen test kit. The remaining portion was subjected to total RNA extraction and the RNA concentration of each organ tissue was diluted to the unified level followed by cDNA synthesis according to the methods described above. The animal research obtained specific approval and guidance from South China Agriculture University’s Institutional Animal Care and Use Committee. And our animal research in our study had been approved by Guangdong Province Animal Disease Control Center.

### Primer design

The *gp85* gene sequences of ALV-A/B strains aligned using DNAStar (DNASTAR, Inc.,Madison) were retrieved from the GenBank database (GenBank acc. nos. M37980, M19113, L10922.1, HM775328, HM452341, HM452342, HM452339, HM452340, DQ365814, M14902 and HM446005) and the full-length proviral genome sequence of the isolate GD13-1 [23]. Only a highly conserved region was used to design primers for the multiplex detection of avian leukosis virus subgroups A and B. To detect and distinguish ALV-J from other chicken ALV subgroups (ALV-A, ALV-B, ALV-C, ALV-D, and ALV-E), a pair of primers was designed by aligning the *gp*85 gene sequences of ALV-A/B strains described above and previously published ALV sequences including ALV-C: PragueC (J02342), ALV-E:RAV-0(M12172), ALV-J:HPRS103(Z46390), NX0101(DQ115805), HN06(HQ900844). The primer sequences of GAPDH were designed based on a conserved region of chicken GAPDH gene (NM_204305). The optimal primers (Table 6) were syntheszsed by Invitrogen (Shanghai, China).

**Table 6:** **Primers used in this study**

**Purpose**	**Name**	**Sequence (5′-3′)**	**Amplicon size (bp)**
env gene amplification of ALV- A/B strains	A/B-F	CACGGTTCCTCCTTAGACA	242
	A/B-R	CCGATTAACCCATATCCCTCC	
env gene amplification of ALV J strain	J-F	TGTGTGCGTGGTTATTATTTC	144
	J-R	AATGGCGAGGTCGCTGACTGC	
GAPDH gene amplification of chicken	G-F	GAACATCATCCCAGCGTCCA	112
	G-R	CGGCAGGTCAGGTCAACAAC	

### Real-time PCR

Real-time PCR were performed on an ABI 7500 Real-time PCR System (Applied Biosystems) using iQ SYBR Green Supermix reagents (BIO-RAD) according to the Manufacturer’s specification. The reaction was performed in a 20 μL system containing 10 μL 2 × iQ SYBR Green Supermix, 0.5 μL of 20 μM forward primer, 0.5 μL of 20 μM reverse primer, 0.8 μL of cDNA and 8.2 μL double-distilled water (ddH_2_O). The real-time PCR was carried out as follows: 1 cycle of 95°C for 5 min, followed by 40 cycles of 95°C for 15 s and 60°C for 35 s. Fluorescent signals were collected during the elongation step.

### Plasmid standard preparation

To construct a recombinant plasmid containing the env and GAPDH genes for establishing a standard curve, conventional RT-PCR amplification of the env genes from ALV-A GD13-1 cDNA , ALV-B CD08 cDNA , ALV-J CHN06 cDNA and GAPDH gene from DF-1 cell cDNA were carried out respectively and the primers used are listed in Table 6. Following amplification, the PCR fragments were cloned into the pMD-18 T vector (TaKaRa) to obtain the recombinant plasmids pMDA from GD13-1 cDNA, pMDB from CD08 cDNA, pMDJ from CHN06 cDNA and pMDG from DF-1 cell cDNA. The plasmid concentration was determined with the ND-1000 spectrophotometer (NanoDrop), and the copy number was calculated using the following formula: number of copies = (concentration in ng × 6.02 × 10^23^)/(genome length × 10^9^ × 650) [24]. The recombinant plasmids (10^10^ copies/μL) were 10-fold serially diluted with ddH_2_O and used for the construction of standard curves.

### Routine PCR

Routine PCR amplification of different subgroups exogenous ALVs from GD13-1 cDNA , CD08 cDNA and CHN06 cDNA were performed with the subgroup-specific primers described in Table 7 [25-27]. Subsequently, the PCR product was purified to construct a recombinant plasmid according to the methods described above. The sensitivity of this conventional RT-PCR assay was evaluated with the newly constructed plasmid diluted serially 10-fold and compared with those of Real-time PCR assay.

**Table 7:** **Primers employed to detect ALV**

**Virus**	**Primer**	**Primer sequence(5′-3′)**	**Size of PCR product**
ALV-A [25]	F: H5	GGATGAGGTGACTAAGAAAG	692 bp
	R: capA	AGAGAAAGAGGGGTGTCTAAGGAGA	
ALV-B, D [19]	F: BD-F	CGAGAGTGGCTCGCGAGATGG	1.1 kb
	R: BD-R	AGCCGGACTATCGTATGGGGTAA	
ALV-J [20]	F: H5	GGATGAGGTGACTAAGAAAG	545 bp
	R: H7	CGAACCAAAGGTAACACACG	

### Statistical analysis

Statistical comparisons were made by GraphPad Prism 5 (GraphPad Software Inc., San Diego, CA) and statistical significance was represented by P values of >0.05, <0.05, 0.01 or 0.001.
